# Maximizing Explanatory Power in Stereological Data Collection: A Protocol for Reliably Integrating Optical Fractionator and Multiple Immunofluorescence Techniques

**DOI:** 10.3389/fnana.2018.00073

**Published:** 2018-10-30

**Authors:** Anna Kreutz, Nicole Barger

**Affiliations:** ^1^Neuroscience Graduate Program, University of California, Davis, Davis, CA, United States; ^2^Department of Psychiatry and Behavioral Sciences, University of California, Davis, Sacramento, CA, United States; ^3^MIND Institute, University of California, Davis, Sacramento, CA, United States

**Keywords:** optical fractionator, photobleaching, Ctip2, NeuN, neurons, microglia, oligodendrocytes, astrocytes

## Abstract

With the promise of greater reliability and replicability of estimates, stereological techniques have revolutionized data collection in the neurosciences. At the same time, improvements in immunohistochemistry and fluorescence imaging technologies have facilitated easy application of immunofluorescence protocols, allowing for isolation of multiple target proteins in one tissue sample. Combining multiple immunofluorescence labeling with stereological data collection can provide a powerful tool to maximize explanatory power and efficiency, while minimizing tissue use. Multiple cell classes, subtypes of larger populations, or different cell states can be quantified in one case and even in one sampling run. Here, we present a protocol integrating stereological data collection and multiple immunofluorescence using commonly employed widefield epifluorescence filter sets, optimized for blue (DAPI), green (FITC), and far red (CY5) channels. Our stereological protocol has been designed to accommodate the challenges of fluorescence imaging to overcome limitations like fixed filter sets, photobleaching, and uneven immunolabeling. To enhance fluorescence signal for stereological sampling, our immunolabeling protocol utilizes both high temperature antigen retrieval to improve primary antibody binding and secondary antibodies conjugated to optimally stable fluorophores. To illustrate the utility of this approach, we estimated the number of Ctip2 immunoreactive subcerebral projection neurons and NeuN immunoreactive neurons in rat cerebral cortex at postnatal day 10. We used DAPI (blue) to define the neocortex, anti-NeuN (far red) to identify neurons, and co-labeling of anti-Ctip2 (green) and anti-NeuN (far red) to isolate only subcerebral projection neurons. Our protocol resulted in estimates with low sampling error (CE < 0.05) and high intrarater reliability (ICC > 0.98) that fall within the range of published values, attesting to its efficacy. We show our immunofluorescence techniques can be used to reliably identify other cell types, e.g., different glial cell classes, to highlight the broader applications of our approach. The flexibility of the technique, increasingly reduced costs of fluorescence technologies, and savings in experimental time and tissue use make this approach valuable for neuroscientists interested in incorporating stereology to ask precise neurophysiological and neuroanatomical questions.

## Introduction

The introduction of stereological methods to neuroscientific questions has provided novel and, more importantly, reliable tools for quantitative data collection, rapidly becoming the gold standard in the field. The optical fractionator technique, specifically, is used to obtain numerical estimates of cell number (Gundersen, 1986; West, 1993a). Of particular utility for neuroscientists, it is not reliant on structure volume, circumventing potential confounds that can be introduced by a variety of tissue processing artifacts (West et al., 1991). The optical fractionator has been applied in diverse contexts. In the human brain, work by modern neurostereologists has revealed important insights into the number of neurons and glia in a variety of cortical and subcortical brain structures (West, 1993b; Pakkenberg and Gundersen, 1997; Semendeferi et al., 1998, 2001; Uylings et al., 2006; Pelvig et al., 2008), as well as cellular variation in diverse mental disorders (Berretta et al., 2007; Kreczmanski et al., 2007; Camacho et al., 2014; Morgan et al., 2014). Rigorous stereological methods provide a means to estimate biological effects in experimental research and have been used to characterize neuronal variation in animal models of autism, fetal alcohol syndrome, and schizophrenia (Karlsen et al., 2013; Karacay et al., 2015; Lauber et al., 2016). Many analyses use morphological criteria to discriminate between cell types as revealed by traditional stains, like Nissl. While generally useful, morphological criteria cannot always be used to identify every cell type or process of interest in the brain and alternative approaches may be warranted.

To address a broader array of questions, neurostereology can be performed in conjunction with immunohistochemistry to label specific, biologically meaningful “markers” indicative of particular cell types, subclasses, or even states. Employing multiple immunolabeling can further refine and increase the breadth of experimental questions addressed with stereological analysis. A variety of stereological studies have used a single antibody, e.g., against a protein associated with cortical interneurons or serotonergic fibers, to quantify functionally relevant variation in neuronal subtypes across diverse species (Raghanti et al., 2008; Sherwood et al., 2010; Hou et al., 2011; Stimpson et al., 2016). However, protein markers are not always cell specific, limiting the precision and scope of single-labeling approaches. For example, while expression of the transcription factor Ctip2 can be used as a marker to identify subcerebral projection neurons, a small population of other cells are also immunoreactive for Ctip2 (Arlotta et al., 2005). To ensure only neurons expressing Ctip2 are sampled, an additional neuronal marker, like NeuN, is needed (Mullen et al., 1992; Lyck et al., 2007). In such cases, employing multiple immunolabeling with a combination of markers enhances diagnostic precision, improving internal validity. An obvious additional advantage of multiple labeling is the ability to quantify multiple cell types in one tissue series or even stereological sampling run. In rare cases, this can be accomplished with one antibody. For example, we previously reported that we could use nuclear volume to simultaneously stereologically quantify neurons and all glial cell types, if we used a single antibody to segregate microglia whose nuclear volumes overlap with other glia (Morgan et al., 2014). However, most cell types cannot be identified with traditional counterstains. With a standard four-channel epifluorescence setup, up to four separate markers can be used in the same tissue section to quantify diverse cell types and/or clarify markers with complicated expression patterns.

For multiple labeling, fluorescence provides many advantages over enzymatic immunohistochemistry. Generally, they include: a quicker staining protocol, lower reagent cost, and elimination of toxic chemicals commonly used in chromogenic staining, such as DAB (Griswold et al., 1968; Egilsson et al., 1979; Konopaske et al., 2008; Prasad and Richfield, 2010). Although multiple chromogens can be used in enzymatic techniques, fluorophores emitting in individual fluorescence channels provide more distinct and consistent labeling, improving discrimination between antibodies. For stereology, this helps to ensure that the correct cell types are sampled during data collection. Unlike the relatively uniform signal produced by individual fluorophores with standardized spectral properties, labeling in enzymatic immunohistochemistry is highly dependent on incubation conditions during color development. It is particularly sensitive to timing, resulting in variable labeling if consistent parameters are not maintained. This can complicate identification of markers that are naturally variably expressed, e.g., some transcription factors like Ctip2. Because it eliminates dehydration steps and requires briefer section drying time (∼15 min), immunofluorescence labeling results in substantially increased final section thickness compared with enzymatic techniques (Prasad and Richfield, 2010). Thicker sections are preferred for optical fractionator sampling, while it is easier to identify single cells during sampling when tissue height is less compressed. These advantages of immunofluorescence can be harnessed to benefit stereological investigation.

At the same time, immunofluorescence does introduce challenges. Fluorophore selection will likely be limited by the availability of only a few fixed filter sets, as most labs will find it cost prohibitive to have more than a standard set. Inappropriate fluorophore choices could result in “bleed-through” between fluorescence channels that could be interpreted as false positives and reduce the amount of signal that could be visualized. Thus, fluorescence labeling schemes must be optimized to suit fixed spectra, which requires more initial planning than brightfield microscopy. Secondary antibodies can bind to “sticky” regions of tissue, increasing background signal and making it difficult to discriminate low expressing markers. Most importantly, signal can be rapidly lost during data collection as fluorophores photobleach. Some researchers also favor enzymatic immunohistochemistry for stereology as the labeling is maintained nearly indefinitely. To overcome these challenges, we have tested several experimental procedures and fluorophores to develop a reliable working protocol for the stereological quantification of diverse cell types using immunofluorescence.

Here, we present an optimized protocol that is designed to circumvent major issues with immunofluorescence that could impact stereological quantification of cell population estimates with the optical fractionator. To increase epitope availability and enhance fluorescence signal, it uses antigen retrieval (Ramos-Vara and Miller, 2014). To reduce collapse in tissue height during processing and allow for more complete antibody penetration, immunolabeling is performed on free-floating tissue. To avoid excessive photobleaching during data collection, we tested several fluorophores to choose the most photostable fluorophore. As a test of our protocol, we present an optical fractionator study of rat cortex at postnatal day 10 that estimates: (1) the number of subcerebral projection neurons, defined by co-labeling of the established markers Ctip2 and NeuN (Arlotta et al., 2005; Lyck et al., 2007), and (2) the total population of NeuN^+^ neurons. We selected Ctip2 because, as mentioned, it makes for a particularly difficult test case, slowing data collection and increasing the potential for photobleaching. Attesting to the efficacy of our protocol, we found that it facilitated the unambiguous identification of Ctip2^+^/NeuN^+^ subcortical projection neurons, yielded low error rates, and produced estimates of NeuN^+^ neurons consistent with published data, even in a small sample of animals (*N* = 5). To show that our protocol could be extended to address diverse neurobiological questions, we additionally illustrate that various cell types, like microglia, oligodendrocytes, and astroglia, as well as cell states, like “activated” or “quiescent” microglia, can be identified using our immunofluorescence protocol.

## Materials and Equipment

### Multiple Immunolabeling

#### Equipment

Cryostat or microtome

Rotator (Barnstead Lab-Line, 4630).

Stir plate (VWR, 12365-382).

6 qt. rice steamer (Oster, model 5712).

#### Reagents and Solutions

##### Cryoprotectant

30% sucrose in 0.1M PBS (phosphate buffered saline).

##### Refrigerator storage solution

0.01% sodium azide (Acros, 19038-1000) in 0.1M PBS.

##### Tissue collecting solution for freezer storage

Glycerol (Fisher, G33-1).

ddH_2_O (double distilled water).

Ethylene glycol (Fisher, E178-1).

0.2 M PBS.

##### Fluorescence labeling

2–3 primary antibodies from host species with no cross-reactivity (e.g., chicken, goat, and rabbit).

2–3 secondary antibodies from one host species directed against the primary antibody hosts and conjugated to a green, red, or far red fluorophore (e.g., donkey anti-rabbit conjugated to AF-488).

DAPI.

##### 10 mM citrate buffer, pH 6.0

Citric Acid, Anhydrous (Affymetrix, AAJ1372936).

Tween20 (Acros, AC233360010).

ddH_2_O.

##### Antibody dilution buffer

Serum matched to secondary antibody host species (e.g., donkey serum: Millipore, 566460).

Triton X-100 (Acros, AC327372500).

0.1 M PBS.

##### Mounting medium

Glycerol (Fisher, G33-1).

Mowiol (Calbiochem, 475904).

ddH_2_O.

0.2 M Tris Buffer, pH 8.5.

#### Materials

24-well plates or Eppendorf tubes for tissue storage.

Netwells in 12-well plates (Corning, 3478).

Heat-resistant plastic jars (Histoplex).

Superfrost Plus Glass Slides (Fisher, 12-550-15).

Coverslips, 0.13–0.17 mm (Fisher, 12-548-5p).

Optimal Cutting Temperature (Fisher, 23-730-571).

Hooked glass rod or brush to manipulate tissue.

Brain tissue previously fixed with 4% Paraformaldehyde or 10% Formalin.

### Optical Fractionator

#### Equipment

Stereology software suite (Stereo Investigator: MBF Bioscience, Williston, VT, United States).

Computer.

Microscope (Olympus BX61 microscope: Olympus, Tokyo, Japan).

High magnification oil lens, numerical aperture > 1.0 (60× PlanApo: Olympus, Tokyo, Japan).

Low magnification air lens (2× PlanApo: Olympus, Tokyo, Japan).

Fluorescence illumination system (Prior, Rockland, MA, United States).

Filter Cubes (DAPI, FITC, TRITC, and Cy5 filter sets: Chroma, Bellows Falls, VT, United States).

Monochrome video camera with high sensitivity in visible and near infra-red wavelengths (e.g., Hamamatsu, ORCA-ER-1394).

Automated stage (Prior, Rockland, MA, United States).

Microcator (Heidenhain, Plymouth, MN, United States).

#### Reagents and Solutions

Immersion oil, refraction index matched to mounting medium (e.g., Olympus, MOIL-30).

#### Materials

Immunolabeled tissue series.

## Stepwise Procedures

### Sectioning

For the optical fractionator, tissue should be cut in a consistent manner, maintaining a common section thickness. While the optimal sectioning method is debated, measures can be incorporated into stereological study design to buffer against biases introduced by specific processing techniques (Dorph-Petersen et al., 2001; Gardella et al., 2003; Schmitz and Hof, 2005). Especially, application of the estimators introduced by Dorph-Petersen et al. (2001) and described in Section 3.3 will produce the most reliable estimates across sectioning techniques. We choose to cryosection tissue at 50 μm to ensure tissue thickness after shrinkage does not fall below the recommended minimum of 15 μm for counting neurons (Howard and Reed, 2005). Our immunofluorescence protocol can mitigate the influence of potential sources of bias reported for cryosectioned tissue, namely, poor cell morphology and considerable collapse in thickness (Dorph-Petersen et al., 2001; Ward et al., 2008). Cell identification is enhanced by fluorescent-tagged antibodies and shrinkage is reduced by omitting dehydration steps and minimizing exposure to air. As alternatives, celloidin embedding is impractical for multiple immunolabeling and paraffin embedded tissue requires considerably more processing, although they are reported to produce good numerical estimates with Nissl stains (Gardella et al., 2003; Ward et al., 2008). Additional processing steps can introduce more opportunities for failed or inconsistent immunolabeling across individual tissue series. Vibratome sectioning has been indicated to produce some of the most extreme artifacts but may be used with appropriate corrections (Dorph-Petersen et al., 2001; Gardella et al., 2003; Ward et al., 2008). Prior to sectioning, whole brains or tissue blocks should be fixed by immersion or perfusion in accordance with laboratory protocol and university Institutional Animal Care and Use Committee (IACUC) guidelines (for more detail on our perfusion protocol see Cunningham et al., 2013). All brains used in this study (*N* = 5) were perfused. Our study was performed in compliance with the NIH Guide for Care and Use of Laboratory Animals and the University of California at Davis IACUC. Our protocol proceeds as follows.

#### Tissue Processing

(A) Transfer a whole brain or tissue block to a cryoprotectant solution containing 30% sucrose in 0.1 M PBS. The brain is ready for freezing when it is saturated with cryoprotectant and has sunk to the bottom of the solution.
  • For small brains or tissue blocks, this can take 24 h. For larger brains, it can take longer. In the latter case, solutions should be changed every 3–5 days or 0.01% sodium azide should be added to the solution to prevent contamination.
(B) Freeze brains to prepare for cryosectioning.
  • We freeze small brains in a container housing a glass dish containing 2-methylbutane cooled on dry ice. Enough 2-methylbutane should be added to cover most, but not all, of the cryomold. We extract the specimen from the cryoprotectant and gently dab it on a Kimwipe to remove excess liquid before placing the brain in a cryomold and covering in Optimal Cutting Temperature (OCT). Note the orientation, the front, and the back of the specimen on the cryomold prior to freezing. Once tissue has frozen to a white solid, usually ∼10 min, brains can be stored at -80°C or cut immediately.
(C) Cryosection brains coronally at 50 μm on a cryostat or sliding microtome.
(D) Collect sections, systematically, in a numbered well plate or Eppendorf tubes, ensuring the rostrocaudal order of sectioning is maintained.
  • Well plates and tubes can be filled with 0.01% sodium azide for short term storage at 4°C or tissue collecting solution (TCS) for longer storage at -20 or -80°C.

#### Solutions

Cryoprotectant solutions can be used for storing tissue at -20 or -80°C to prevent freezing. This is particularly advantageous for longer term storage. We use the following TCS as our cryoprotectant.

##### TCS protocol

(A) Prepare a solution containing:
  500 mL Glycerol.
  400 mL ddH_2_O.
  600 mL Ethylene glycol.
  500 mL 0.2 M PBS.
(B) Mix well.
(C) Store at 4°C.

### Immunolabeling

**FIGURE 1:**
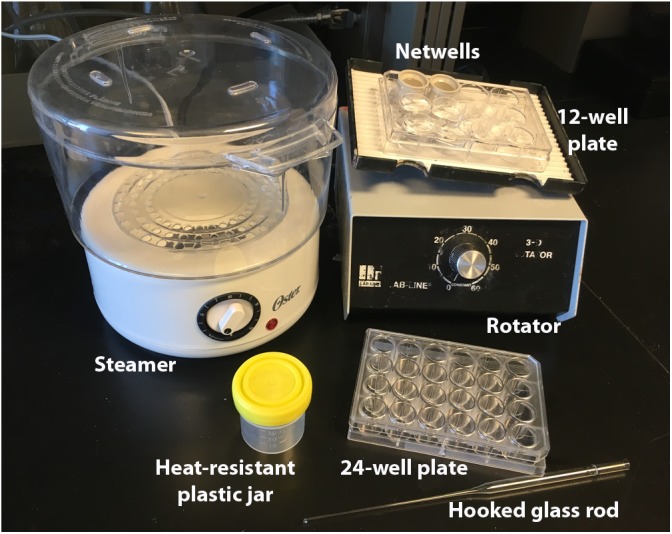
Immunolabeling equipment. Equipment necessary for our antigen-retrieval immunolabeling procedure. Netwells and 12-well plate are used for PBS rinses, steamer and plastic jars for antigen-retrieval, 24-well plate for blocking and antibody steps, and glass rod for manipulating tissue.

For immunolabeling, we employ several modifications intended to increase signal, reduce background fluorescence, and preserve section thickness to support stereological data collection. We use a variant of common heat induced epitope retrieval (HIER) techniques that incorporates a neutral pH Citrate Buffer and rice steamer (Figure 1). Compared with other heating methods, like microwaving tissue, the steamer provides steady, high temperature heating, is inexpensive, produces robust labeling of our antigens of interest, and reduces tissue deformation (Tang et al., 2007; Ramos-Vara and Miller, 2014; Vinod et al., 2016). This allows us to stain tissue free-floating to improve antibody penetration. While high heat antigen retrieval increases the availability of many epitopes, it is important to note that some epitopes are sensitive to heat. In these cases, it may be best to omit the antigen retrieval step or use an alternative, lower temperature, method. We also include TritonX-100 in many solutions to increase membrane permeability, which proves especially beneficial for antibodies targeting epitopes localized to the nucleus like Ctip2 and NeuN. Additionally, using fluorophores conjugated to secondary antibodies, rather than directly to primary antibodies, amplifies the signal and provides greater flexibility, increasing the array of commercial fluorophores available for selection and the combination of primary antibodies that can be used in different channels. One drawback is the potential for higher background fluorescence resulting from non-specific binding of secondary antibodies. To reduce false signal, we incorporate serum from the host of the secondary antibodies into our blocking and antibody dilution buffers at high concentrations. This step improves cell discrimination during stereological data collection.

**FIGURE 2:**
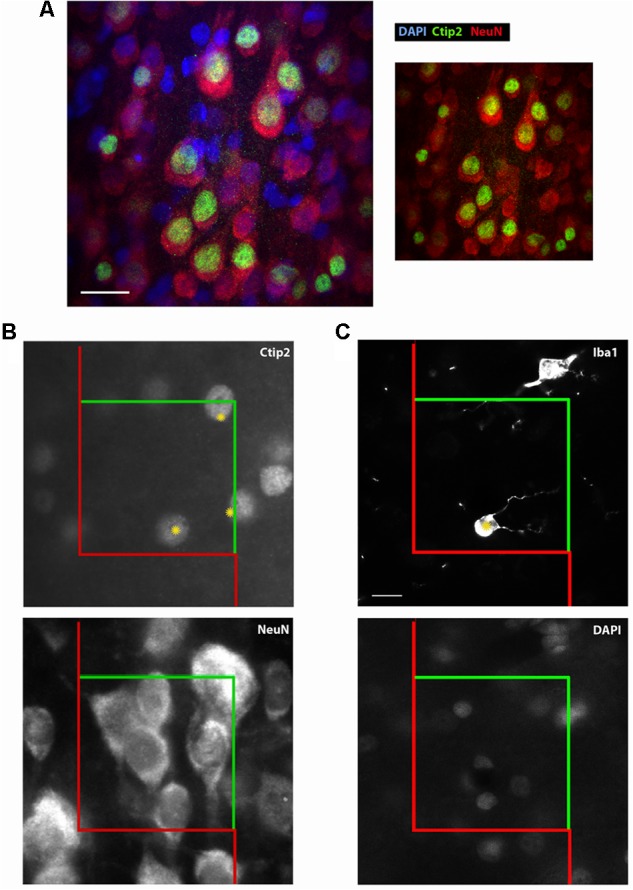
Ctip2 and NeuN labeling. Postnatal day 10 rat cortex immunolabeled with DAPI (blue), Ctip2 (green), and NeuN (far red, colored red here), imaged on our confocal microscope, 60× **(A)**. The transcription factor Ctip2 shows its characteristic nuclear labeling pattern, whereas NeuN labeling is appropriately nuclear and extra-nuclear. Ctip2 is variably expressed in the nuclei of neurons labeled with NeuN. Where Ctip2 expression is low, co-labeling of NeuN, Ctip2, and the nuclear marker DAPI is clear. Ctip2 and NeuN IR in monochrome images at the same sampling site from our epifluorescent setup **(B)**. Ctip2 is in the green channel and NeuN is in the far red channel. Ctip2 and NeuN signals are discrete with no spectral overlap between green and far red channels. Microglia can also be readily quantified using our protocol **(C)**. Microglia can be counted if the widest point of the nucleus, confirmed with DAPI, comes into focus within the counting frame. Scale bar = 20 μm **(A)**, 10 μm **(C)**. Yellow asterisks (^∗^) indicate Ctip2^+^/NeuN^+^ neurons and Iba1^+^ microglia that meet criteria necessary to be sampled in the disector.

**FIGURE 3:**
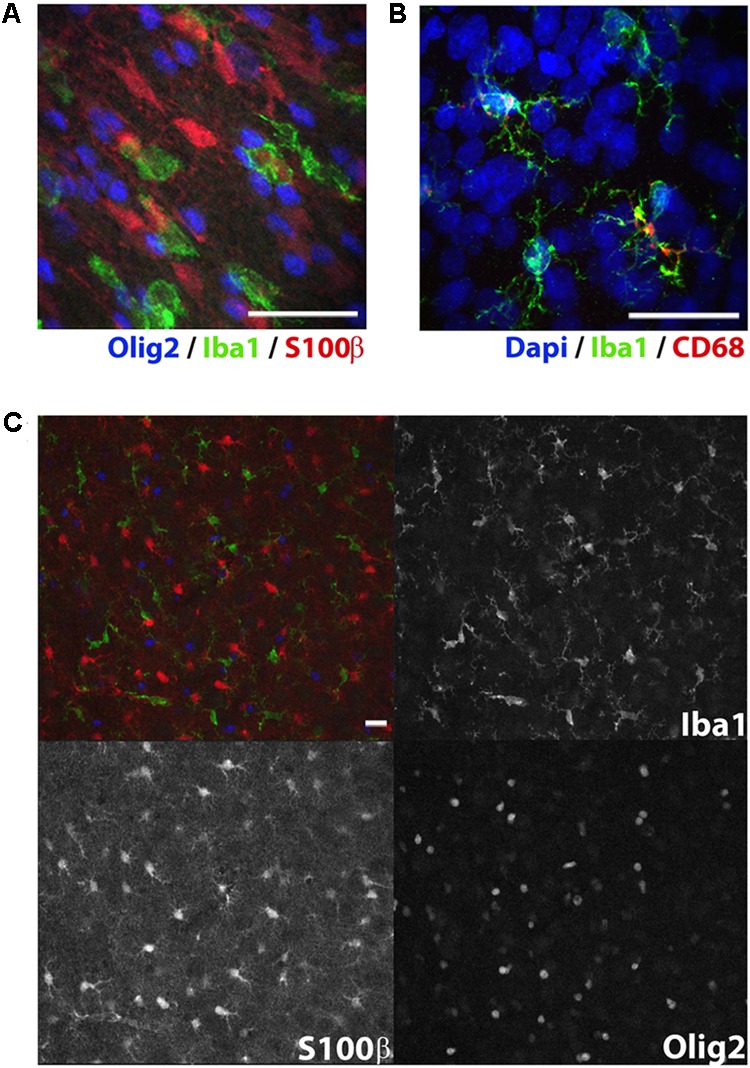
Immunolabeling of glial subtypes in postnatal day 10 rat white matter. Olig2 (far red, colored blue here), Iba1 (green), and S100β (red) clearly label oligodendrocytes, microglia, and astrocytes, respectively **(A)**. Punctate CD68 (red) expression was observed in some but not all Iba1+ microglia (green) in the cerebral cortex **(B)**. Expression pattern of CD68 appropriately reflects the endosomal and lysosomal distribution of this glycoprotein. Expression indicates particularly activated microglia. Low magnification image showing individual channel expression **(C)**. Image acquired with a confocal microscope. Scale bar = 20 μm.

Several considerations need to be taken into account when deciding which secondary antibody conjugated fluorophores to use for multiple labeling. Choosing stable fluorophores with a high quantum efficiency and yield is especially important in widefield epifluorescence microscopy which produces greater light scatter and does not easily filter out of focus photons compared with confocal microscopy (Murphy and Davidson, 2013). Determining which markers to place in which channel is another important consideration. Because they cannot be optimized for every fluorophore, fixed filter sets increase the possibility of fluorescence signal bleed-through between channels. This is particularly problematic when primary antibodies co-localize, increasing the likelihood that bleed-through from adjacent fluorescence channels could be misinterpreted as true co-labeling. It may be necessary to separate these markers into non-adjacent fluorescence channels that would not be excited by the same wavelengths. For our two overlapping markers, NeuN and Ctip2, we were unable to avoid some degree of bleed-through between the green (FITC) and red (TRITC) channels, even after trying multiple combinations of fluorophores. Consequently, we minimized the possibility of spectral overlap by placing them in the green and far red (Cy5) channels, omitting the red channel entirely (Figure 2). Green and red fluorophores tend to be brighter than those in far red and ultraviolet wavelengths (Murphy and Davidson, 2013). To maximize signal from the variably expressed Ctip2 antibody, we used a secondary antibody conjugated to the bright green fluorophore, AF-488, while we labeled the robustly expressed NeuN primary with a dimmer far red secondary, AF-647. All four fluorescence channels could be utilized in cases where antibody targets are easier to discriminate, e.g., when different antibodies label separate, non-overlapping cellular components. For example, we used anti-Olig2 to label oligodendrocyte nuclei in the far red channel with AF-647, anti-Iba1 to label the cell bodies and the fine processes of microglia in the green channel with AF-488, and anti-S100β to label astrocyte cytoplasm and processes in the red channel with AF-594 (Figure 3), finding them to all to be morphologically discriminable (Dyck et al., 1993; Ito et al., 1998; Zhou and Anderson, 2002).

A primary disadvantage of fluorescence stereology is photobleaching. In order to overcome this issue, a number of parameters can be optimized, most critically the specific fluorophores used. Prior to running the stereological analysis, we suggest verifying the robustness of labeling through photobleaching experiments to identify which fluorophores would be sufficiently stable. We tested secondary antibodies conjugated to green, Cy2 (Jackson) and AF-488 (Life Technologies), and far red, AF-647 (Jackson) and NL-637 (R&D Systems), fluorophores directed against our primary antibody, rabbit-anti Ctip2, each at a dilution of 1:500. Another parameter that can be optimized to minimize photobleaching is mounting media. We prepared slides with one fluorophore, AF-488, coverslipping with either our own mounting medium, Mowiol, or one sold as being particularly stable, Prolong Gold. All slides were allowed to dry overnight. We stimulated each fluorophore with the appropriate filter for up to 15 min, taking images of fluorescence emission after 0.5, 2, 3, 4, 5, 6, 8, 10, 12, and 15 min. Exposure time was set at 250 ms to give a relatively bright, even image of Ctip2 across samples. Using images from our set exposure times, we then determined the mean gray values in Fiji (ImageJ) and calculated the percent reduction in signal intensity. We subsequently chose to label Ctip2 with AF-488 and NeuN with AF-647 and to coverslip with Mowiol because emission of these fluorophores lasted well past the time we anticipated it would take to complete sampling at one probe site during stereological quantification (∼2 min).

All antibodies should be tested on a few sections prior to running the whole series for the analysis, employing proper positive and/or negative controls. Once the appropriate labeling scheme has been established, a series of 10 or more sections through the entire region of interest should be selected from each case for immunolabeling and stereological sampling. According to the fractionator principle (Gundersen et al., 1988), sections should be sampled at evenly spaced intervals with the starting section randomly selected from the first interval for each individual in the analysis. For example, if 40 sections span the region of interest in an individual, immunohistochemistry could be performed on every 4th section to yield 10 sections for stereological analysis. If the random number 2 is chosen as the starting point in the first interval, the sections sampled would include 2, 6, 10, 14, etc. Choosing an even section interval is advantageous because, if the first staining run does not yield a sufficient number of sections through the region of interest, an additional intermediate series can be run to add to existing counts. Our immunolabeling protocol is described below, followed by a step by step protocol for mixing solutions. Table 1 lists concentrations for the primary and secondary antibodies used to test our protocol.

**Table 1:** Overview of primary and secondary antibodies.

Antibody	Dilution	Supplier	Cat. No.
Rabbit anti-Ctip2	1:400	Abcam	ab28448
Rabbit anti-Iba1	1:500	Wako	019-19741
Mouse anti-NeuN	1:400	Millipore	MAB377
Mouse Anti-CD68	1:500	Bio-Rad	MCA341GA
Mouse Anti-S100ß	1:200	Abcam	ab4066
Goat Anti-Olig2	1:500	R&D	AF2418-SP
Donkey anti-rabbit Alexa Fluor-488	1:500	Invitrogen	A-21206
Donkey anti-rabbit Cy2	1:500	Jackson ImmunoResearch	711-225-152
Donkey anti-mouse AF-594	1:500	Jackson ImmunoResearch	715-585-020
Donkey anti-rabbit Alexa Fluor-647	1:500	Jackson ImmunoResearch	711-605-152
Donkey anti-rabbit NL-637	1:500	R&D Systems	NL008
Donkey anti-goat AF-647	1:500	Jackson ImmunoResearch	705-605-147
DAPI	1:1000	Roche	10236276001


#### Antigen Retrieval

(A) Transfer selected sections to netwells in 12 well plates (Figure 1), being careful to track brains run in parallel with careful labeling throughout.
(B) Fill reservoir of the rice steamer with ddH_2_0, replace steamer basket, and rotate knob to 45 min mark.
(C) While waiting for steamer to warm, rinse tissue 3 times for 5 mins in fresh 0.1 M PBS (3 × 5 mins) on a rotator using netwells in plastic 12 well plates.
(D) With a glass hook, transfer tissue to heat-resistant plastic jars filled with 20 mL of 10 mM Citrate Buffer. Cap lids loosely to prevent pressure buildup and place jars around the edges inside the steamer to ensure more even heating.
(E) Heat sections in the steamer for 8–15 min, depending on tissue integrity.
(F) Remove jars, uncap, and allow to cool for 5 min or until room temperature is reached.
(G) Rinse tissue 3 × 5 min in PBS using netwells and 12 well plates on a rotator.

#### Blocking

(A) Rinse tissue 3 × 5 min in PBS using netwells and 12 well plates on a rotator.
(B) Pipette 500 μl of the Blocking Buffer into each well of a 24 well plate.
(C) Transfer sections to well plates with a glass hook being careful to maintain section order.
(D) Place well plate on a rotator at room temperature for 1 h.

#### Primary Antibodies

(A) Add all primary antibodies (up to four, each from different species) to Antibody Dilution Buffer at room temperature.
(B) Pipette 500 μl of the primary antibody solution into each well of a 24 well plate.
(C) Transfer sections to primary antibody solutions with a glass hook being careful to maintain section order.
(D) Incubate sections overnight at room temperature on a shaker.
(E) Rinse tissue 3 × 5 min in PBS using netwells and 12 well plates on a rotator.

#### Secondary Antibodies

(A) Add all secondary antibodies for each of the primary antibodies added in 3.2.3A to Antibody Dilution Buffer.
(B) Pipette 500 μl of the secondary antibody solution into each well of a 24 well plate.
(C) Transfer sections to secondary antibody solutions with a glass hook being careful to maintain section order.
(D) Incubate for 2 h at room temperature on a shaker.
(E) Rinse tissue 3 × 5 min in 1/3 PBS diluted in ddH_2_O using netwells and 12 well plates on a rotator.
(F) Mount tissue onto glass slides in 1/3 PBS.
(G) Coverslip within 15 min of mounting using Mowiol.

#### Solutions

Prepare the following prior to immunostaining. Citrate Buffer and Mowiol can be prepared well in advance and stored as indicated. Blocking and antibody dilution solutions can be prepared during the immunostaining protocol in advance of use. They should incorporate serum from the host of the secondary antibodies. We prefer secondary antibodies raised in donkey because they are commonly available conjugated to an array of different fluorophores.

##### 10 mM citrate buffer, pH 6.0

(A) Add 1.92 g Citric Acid Anhydrous to 900 mL ddH_2_O.
(B) Stir until dissolved.
(C) Adjust pH to 6.0 with NaOH.
(D) Volumize to 1 L.
(E) Add 0.5 mL Tween20.
(F) Store at 4°C for up to 1 month.

##### Mowiol mounting medium

(A) Add 19 mL (24 g) Glycerol, 9.6 g Mowiol 4–88, and 48 mL 0.2M Tris Buffer (pH 8.5) to 24 mL ddH_2_O.
(B) Stir on hot plate at mid-high setting until combined (∼4–5 h) and do not let boil.
(C) Aliquot into 50 mL Falcon tubes.
(D) Centrifuge at 50,000 × *g* for 15 min.
(E) Discard pellet.
(F) Aliquot supernatant.
(G) Store at 4°C for 1 month or -20°C for 12 months.

##### Blocking buffer

(A) Thaw donkey serum on ice.
(B) For blocking, prepare a solution that includes 10% donkey serum and 0.3% Triton X-100.
  • It is helpful to work with a stock solution of 5–10% Triton X-100 to minimize inaccurate pipetting and facilitate mixing of the viscous stock solution.
(C) Volumize with PBS.
(D) Store on ice until use.

##### Antibody dilution buffer

(A) Prepare a solution comprising 8% donkey serum, 0.3% Triton X-100, and PBS.
  • This buffer is used for primary and secondary antibodies.
(B) Store on ice until use.

### Optical Fractionator Data Collection

**FIGURE 4:**
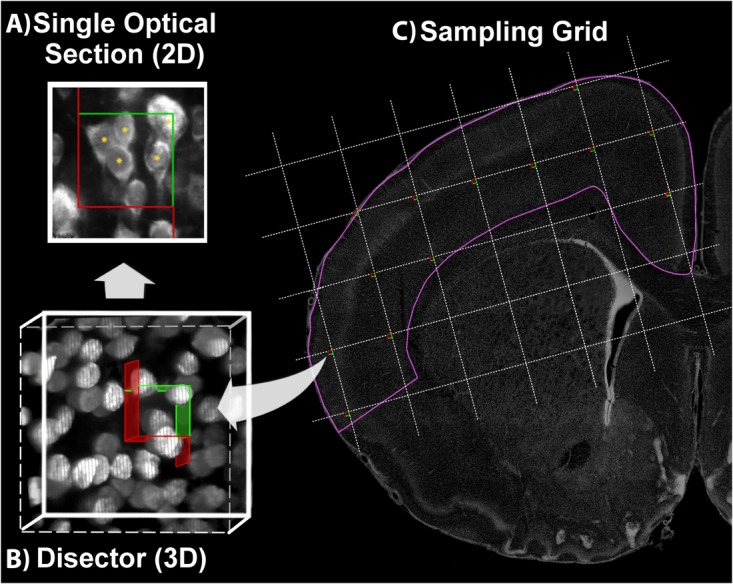
Optical Fractionator. Using a defined criterion (here, the widest point of the Ctip2^+^ nucleus), individual cells in a single optical plane or “section” are marked in the sample (yellow asterisk) if they fall within the disector, touch the green lines of inclusion, and do not touch the red lines of exclusion **(A)**. In the single optical section illustrated here, five cells are counted, including four with nuclei that fall within the disector and one that touches the upper and right green lines of inclusion. One cell is excluded because its lower boundary touches the bottom red line. Cells are sampled in a 3-dimensional disector probe **(B)**. To sample cells, the observer focuses up and down through the entire disector, evaluating each cell that comes into focus in a single optical plane. These 3-dimensional disectors are located at each intersection point on a sampling grid superimposed over each section. This is illustrated on a coronal section of a postnatal day 10 rat brain **(C)**. DAPI was used to delineate the cortical region of interest (pink line). The probe proceeds across the sampling grids from disector to disector in each section until all sections have been sampled.

The optical fractionator is a multi-stage systematic random sampling scheme (Gundersen, 1986; Gundersen et al., 1988; West, 1993a). Decisions about three critical sampling parameters need to be made prior to undertaking the full experimental run: disector volume, grid size, and section sampling interval. The section sampling interval should be chosen prior to immunolabeling as indicated in the previous section, with sections evenly spaced and the first section chosen randomly from the first interval according to the fractionator principle (Gundersen et al., 1988). The disector is a three-dimensional counting frame (box) used to directly sample cells in conjunction with a precise, “dimensionless” criterion identifiable in a single, thin optical plane (Figure 4). Total cell counts are obtained by systematically sampling cells in a series of disectors distributed across all sections in the sample containing the region of interest. These disectors are evenly spaced, located at the intersection of the horizontal and vertical lines of a two-dimensional sampling grid superimposed in a random position over each section. Total cell population (*N*) estimates are derived using the formula:

(1) $$N=\sum Q^{-}\times \frac{1}{ssf}\times \frac{1}{asf}\times \frac{t}{h}$$

where *Q*^-^ is the total number of cells sampled, *t* is the section thickness, and *h* is the height of the disector. The *asf*, or area sampling fraction, is the ratio of the area (length × width) of the disector to the area of the sampling grid square. The *ssf* is the section sampling fraction, or the interval between sampled sections, e.g., 1/10 for every 10th section. While most measures are straightforward, there is some discussion of the appropriate measure for *t*. Cut thickness could be used to approximate section thickness. However, because tissue processing often results in shrinkage and/or other distortions in section thickness, using cut thickness in the equation is not recommended. Using the mean tissue thickness measured across sampling sites provides a better estimate for t. In cases where measured tissue thickness within individual sections is highly variable, it should be number-weighted (for calculation see the original formula proposed by Dorph-Petersen et al., 2001). As a rule, it is best to use number-weighted thickness in the calculation because it provides the most accurate estimates across sampling conditions (Dorph-Petersen et al., 2001; Bermejo et al., 2003).

**FIGURE 5:**
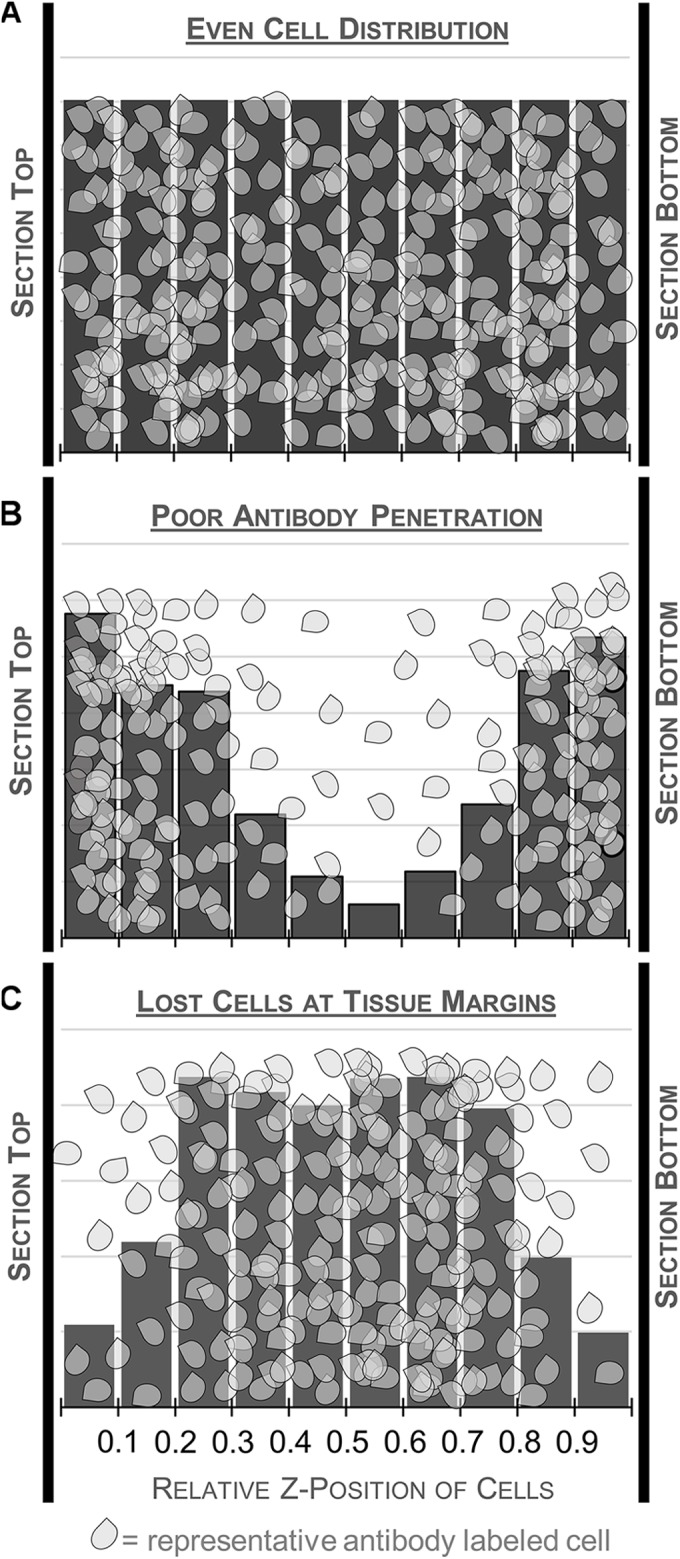
Schematic illustrating different patterns of cell distribution through the tissue thickness as revealed by histograms charting the relative z-position of sampled cells across 10 bins. A relatively even distribution of cells throughout the thickness is ideal and suggests minimal impact of tissue processing (top, **A**). Fewer cells in the middle of the section could indicate poor antibody penetration (middle, **B**). This may warrant processing an additional series from the same case, changing variables that could affect antibody penetration. Histograms with fewer cells at the top and bottom of the tissue suggest cutting artifacts like plucked cells and lost caps may affect the distribution (bottom, **C**). Guard zones can be set to exclude these variable regions.

Guard zones, fixed buffers set above and/or below the disector, should be employed as an additional precaution to mitigate the effects of tissue processing. Processing artifacts at the cut surface, like compression or “lost caps,” can bias cell distribution at the outer margins of the tissue, which could, in turn, bias final estimates (Andersen and Gundersen, 1999; Mouton, 2002; Gardella et al., 2003; Howard and Reed, 2005; Ward et al., 2008). Thus, it is often advisable to situate the disector away from the cut surface, where cell distribution in the z-axis is relatively uniform and less affected by processing, to ensure accurate estimates (Figure 5). For example, the top of the disector is commonly set at least 2.5 μm below the top of the tissue section, i.e., with a 2.5 μm guard zone. In some cases, complete omission of guard zones has been advocated (Hatton and von Bartheld, 1999; Carlo and Stevens, 2011). We suggest that a preliminary analysis of cell distribution in the z-axis be performed to evaluate these factors.

**FIGURE 6:**
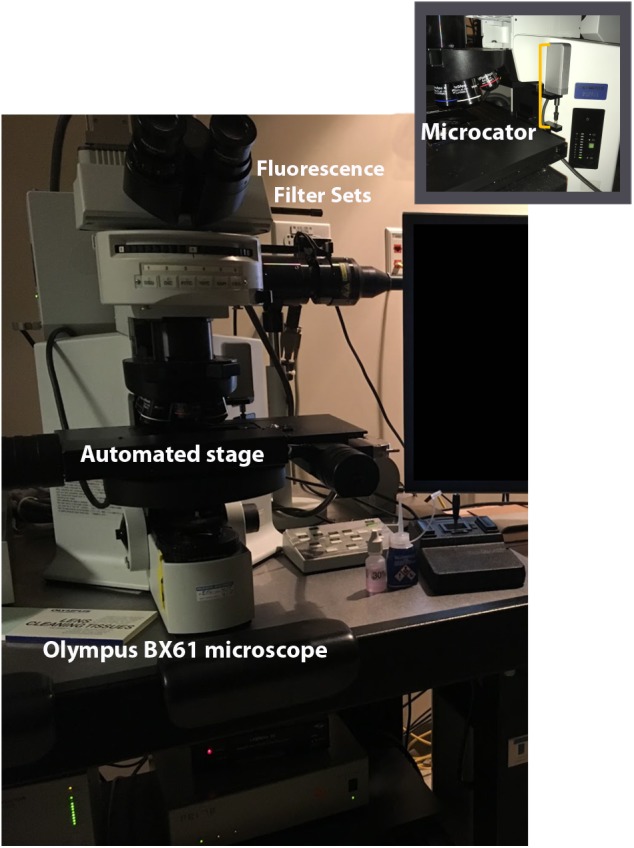
Stereology setup. Computer workstation with Stereo Investigator is attached to the microscope. Microscope includes an automated stage and microcator for measuring *z* depth. Fluorescent filter sets allow for viewing multiple channels. Illumination is provided by an epifluorescent illumination system (not shown).

Our analyses are performed using a widefield epifluorescence Olympus BX61 microscope (Olympus, Tokyo, Japan) and the Stereo Investigator (MBF Bioscience, Williston, VT, United States) software suite. In addition to the standard stereology setup requiring an automated stage (Prior, Rockland, MA, United States) for systematic sampling and microcator for measuring tissue thickness (Heidenhain, Plymouth, MN, United States), the microscope (Figure 6) is equipped with a Lumen 200 fluorescence illumination system (Prior, Rockland, MA, United States) with filter cubes optimized for DAPI (blue), FITC (green), TRITC (red), and Cy5 (far red) (Chroma, Bellows Falls, VT, United States). Fluorescence signal is transmitted to a Dell workstation via a monochrome video camera with high sensitivity in visible and near infra-red wavelengths (Hamamatsu, ORCA-ER-1394), which improves visualization and reduces fluorophore fatigue. The light source houses a mercury bulb, but less expensive LED-based units are increasingly popular. We use a 60× Plan Apochromat oil immersion lens (NA, 1.42) for sampling because it is corrected for variation in multiple spectral wavelengths and the high numerical aperture allows for fine optical sectioning (North, 2006; Smith, 2011).

Although the minimum suggested number of counts for stereological estimates is 100–200, we employ a design with an increased sampling intensity because it improves accuracy and can be done without a substantial increase in time spent sampling (Gundersen et al., 1999; Schmitz and Hof, 2000; Slomianka and West, 2005). Moreover, this ensures that cases at the lower end of natural variation will not be thrown out due to insufficient counts from more generous sampling parameters. This buffer is especially important when measuring pathological effects that may produce variation that cannot be anticipated. To produce reliable estimates of cell population number using optical fractionator, we initially determine appropriate sampling parameters on a subsample of cases, attempting to minimize error. Below, we describe our procedure for data collection and illustrate its application on a pilot analysis of neurons, defined as NeuN^+^ cells labeled with the far red fluorophore AF-647, and subcerebral projection neurons, defined as NeuN^+^/Ctip2^+^ cells additionally co-labeled with the green fluorophore AF-488, in postnatal day 10 rat cerebral cortex (*N* = 5).

#### Determine Sampling Parameters

(A) Delineate the region of interest at low power (1–4× objective) in 2–3 individual sections each for a small sample of cases (Figure 4).
  • In multiple immunofluorescence, boundaries can be defined using DAPI to reveal cytoarchitecture or a cell specific marker characteristic of the region. Because our region of interest is the cerebral cortex, we could use the distribution of NeuN labeled neurons in the far-red channel to define this area. However, we chose to use DAPI because it is equally reliable for our structure of interest and allowed us to preserve the more photosensitive NeuN AF-647 signal for stereological sampling.
  • Use a precise anatomical definition to ensure the region is consistently delineated. We defined the neocortices to include all regions of the cerebral cortex except regions comprising four or fewer layers like the hippocampal, olfactory, and amygdalar cortical territories traditionally considered part of the allocortex (Altman et al., 1973; Reep, 1984; Bayer and Altman, 1993; Wise, 2008; Zilles and Amunts, 2012).
(B) Perform a preliminary survey of tissue thickness at several sampling sites within each section delineated in A.
  • DAPI can be used for thickness measurements because it is evenly distributed throughout the thickness of the tissue and less critical for stereological analysis.
  • A high NA lens (NA > 1) with refractive index matched to the mounting medium should be used for fine optical sectioning and submicron level precision. We use a 60× Plan Apochromat oil immersion lens (NA, 1.42) and final magnification of 600×.
  • To measure thickness, set the 0 point as the top of the section, the focal plane where the top of the first visible DAPI labeled cell (or cells) just comes into focus.
  • Focus down through the tissue to the plane where the bottom of the last cell (or cells) is visible and record that z-position as the section thickness at that site.
  • Continue sampling a few sites (∼5–10) on each delineated section and record thicknesses at each site.
  • Data can be recorded in a spreadsheet program to determine average, minimum, and maximum thicknesses and variability across sites.
  • We use tools in Stereo Investigator to track our measurements, but these measurements can be done on any microscopy system that includes a *z*-axis depth gauge (microcator) as previously described elsewhere (West et al., 1991; Mouton, 2002; Williams et al., 2003; Howard and Reed, 2005; Schmitz and Hof, 2005).
(C) Some authors advocate measuring the distribution of immunolabeled cells through the depth of the tissue thickness in a representative set of sites (e.g., Ward et al., 2008). This can reveal the impact of processing artifacts at the tissue’s margins and also provides a means to check antibody penetration (Figure 5) to meet the requirement that every cell has an equal chance of being sampled in the counting frame. Because this exercise can be time consuming and is not considered standard practice in every lab, we present it as an optional step intended to provide additional information for initially determining the sampling scheme. It does not necessarily need to be performed before every stereology experiment (Williams et al., 2003), but instead should be performed judiciously, for example, when using a new sectioning method or antibody.
  • This can be easily accomplished in Stereo Investigator by starting an optical fractionator probe run with the section thickness set at the maximum measured thickness from Step B and a large grid size to produce a small number of sampling sites per section. See Data Collection C-F for sampling method. If the file is exported to Excel, *z*-axis values, thickness, and *z* distribution are computed in the exported spreadsheet. For a detailed protocol for sampling without Stereo Investigator see Williams et al. (2003).
  • Using the same setup as in B, find the top and bottom of the section using DAPI and record section thickness.
  • Switch channels and focus on the top of the first visible immunolabeled cell in the counting frame and mark its position.
  • Continue focusing down through the tissue and mark the *z*-position of each labeled cell as it comes into focus.
  • Switch fluorescence channels and perform the same exercise for the next set of labeled cells.
  • Continue to sample 5–10 sites in each section delineated in A.
  • Once a representative sample (∼200–300 cells) is taken for each antibody, enter the results into a spreadsheet or statistical software program.
  • For each site, calculate the relative *z*-position of each cell by dividing the *z*-position by the total tissue thickness measured at that site. For example, if a cell is located 2 μm below the top of a 20 μm section, its relative position would be 0.10.
  • With these standardized values, a frequency distribution can then be calculated with the pooled relative thickness measures across sites, e.g., using the histogram function in Excel across 10 bins of relative tissue depth (Figure 5). Graphing the frequency distribution can help to identify processing artifacts that may influence final results. Cells should be relatively evenly distributed in the histogram, indicated by a flat distribution. Histograms indicating higher densities at the top and bottom of the tissue could suggest tissue compression during processing or poor antibody penetration. Low densities at the section margins likely result from blade artifacts during cutting, like plucked cells and lost caps.
(D) Determine appropriate disector and guard zone height for the sample based on information obtained in steps B and C.
  • As a reference, a disector height of 9 μm and guard zone of height 2.5 μm have been recommended as optimal minimum values for neurons and similarly sized cells (Howard and Reed, 2005) but values should be evaluated empirically. Disector height plus upper and lower guard zone height should not exceed the thickness of the thinnest sampling site measured in B.
  • Guard zones should be set to exclude any exceptionally dense or sparse areas at the margins of the tissue revealed in the frequency distribution graph from C.
  • When step C is performed, the best effort should be made to set guard zones so that the disector is situated where cell distribution is most even, i.e., by manipulating guard zone height, to avoid sectioning artifacts.
(E) Set disector length and width to ensure approximately 1–3 particles, usually cells, are counted per disector.
  • This parameter will vary by cell density. It can be determined experimentally by starting an optical fractionator run and testing different dimensions on a few sections. See Data Collection C-F for sampling method.
(F) Set up the sampling grid, aiming for a grid size (step size) that yields approximately 200–500 sampled particles per run.
  • Recommendations for the appropriate sample size range between 100 (West, 1993a) and 1000 (Slomianka and West, 2005). 200 particles (cells) is a commonly recommended standard. Statistically, increasing sample size should increase accuracy but sampling efficiency must also be considered in the stereological design. We find aiming for 500 cells per region per case yields very low error rates and can be done without adding excessively to time spent sampling.
  • Assuming 1–3 cells per sampling site per disector, determine the grid size needed to produce a total of 100–200 sampling sites across an entire case based on the traced sections.

#### Data Collection

(A) In Stereo Investigator, we first create a new case using the Optical Fractionator Workflow function. Sections are set up in the serial section manager, indicating the cut section thickness and interval between sections.
(B) Delineate the region of interest at low magnification on the first slide as in Determine Sampling Parameters A.
  • A small, unique reference point (e.g., a distinct blood vessel) can be traced on each section at high magnification to facilitate proper alignment for sampling later.
(C) Set criteria for inclusion in the sample.
  • With immunofluorescence, the primary criterion for inclusion is relatively straightforward—antibody reactivity and fluorescent signal in the dedicated channel. Fluorescence signal should be clearly higher than background and should not be present in unlabeled channels, in our case the red channel. Signal with the same pattern in all channels would indicate autofluorescence.
  • Consider co-labeling. We took advantage of fluorescence co-labeling to improve identification of subcerebral projection neurons, counting only Ctip2^+^ cells that were also NeuN^+^.
(D) Once general criteria are set, a unique, “dimensionless” feature that has a high likelihood of only being sampled in one z-plane should be established *a priori*.
  • Both Ctip2 and NeuN antibodies clearly label the nucleus, so we used the nucleus’s widest point as our criterion for inclusion. The single point where the top or bottom of the counting unit come into focus are additional, commonly used criteria. Caution should be taken to determine the influence of overprojection in the *z*-axis on the chosen criterion when working with fluorescent material.
(E) Start the optical fractionator probe run, progressing along the grid from disector to disector to collect sampling data. Each cell type can be counted in the same run using different markers or each in its own run, if the densities are substantially different.
  • In the first disector, mark only cells that meet the above criteria and that come into focus within the disector or intersecting the green lines of inclusion (Figures 2, 4). If they touch the red lines of exclusion, they should not be counted.
  • Ideally, section thickness measurements will be taken at all sites or at even intervals across sampling sites to allow for estimates based on number weighted thickness. Using a ubiquitous nuclear marker like DAPI will ensure the most accurate measurements. In cases where it is not practical to use a nuclear marker, it has been recommended to cross-check readings for the tissue top and bottom under both epifluorescence and low transmitted visible light (Negredo et al., 2004).
(F) Continue until all sections have been sampled.
(G) Determine numerical estimates using Eq. 1 and the appropriate thickness measure.
  • In Stereo Investigator, this is done by selecting all sampled sections in the serial section manager and exporting results for that case to Excel. The output provides a record of the number of cells sampled, number of sampling sites visited, and stereological parameters in addition to population estimates based on multiple alternative thickness measures and error values.

#### Error Estimates

(A) It is also advisable to perform intraobserver reliability tests on a small number of cases to assess the effectiveness of the defined parameters and to reduce experimenter error from inconsistent application of stereological criteria.
  • Prior to the full run, we sample 2–3 cases that are subsequently resampled 2 times by the same rater.
  • To assess reliability, we use the intraclass correlation statistic, comparing total cells counted in each section for each of the three independent runs.
  • We aim for a single measures coefficient of 0.95 or greater, reflecting a high degree of correspondence between counts performed by one rater.
  • Criteria for inclusion are refined until the criterion can be consistently applied to meet this level of reliability.
(B) It has become common practice to report the Gundersen Coefficient of Error (CE) (Gundersen et al., 1999) as a measure of the error in the sampling scheme, although alternative calculations of experimental error are available. Estimators proposed by Geiser et al. (1990), Scheaffer et al. (1995), Cruz-Orive (1999), Schmitz and Hof (2000), and Cruz-Orive and Geiser (2004) are automatically calculated in Stereo Investigator and included with the standard output, while the original calculations can be explored in the cited literature.
  • Two variants of the Gundersen CE have been proposed to assess error, incorporating smoothness measures *m* = 1 or *m* = 0 when sampling regularly or irregularly distributed objects, respectively (Gundersen and Jensen, 1987; Gundersen et al., 1999). A maximum Gundersen CE of 0.10 is commonly accepted as the standard in publication, indicating no more than 10% of total variation is contributed by the stereological design. Our approach produces very low Gundersen CEs, generally under 0.05.
  • While this 10% criterion is commonly employed pragmatically, in theory, acceptable CEs may vary by experiment. It has been suggested that the error introduced by the stereological design (CE) should comprise only a “negligible” amount (Gundersen et al., 1999) and not more than 50% of the total variance in the group analyzed (CV), i.e., CE^2^/CV^2^ < 0.5 (Gundersen and Jensen, 1987), although dynamics in variance within and between sample groups should be considered when applying this rule (Slomianka and West, 2005). In their empirical test of alternative error measures, Slomianka and West (2005) found that a Gundersen CE, *m* = 0, performed well when estimating particle numbers with irregular distribution across a range of sampling frequencies, but also suggest comparing these error rates with Cruz-Orive’s split-sample estimator, as it more directly reflects variability within the sample. They recommended to increase sampling intensity if these two measures disagree. This more stringent test may be particularly warranted in the pilot phase, when determining initial sampling parameters. Also useful in the planning stage, Cruz-Orive et al. (2004) present a method for determining acceptable CEs in the broader experimental context, addressing their relationship to other critical aspects of study design like population variance, statistical power of the overall design, and sample size.
  • An additional measure suggested to reduce sampling error is the smooth fractionator design, an alternative sampling scheme intended to minimize error resulting from abrupt changes in particle distribution between sampled sections (Gundersen, 2002). In this method, sections are rank-ordered based on a proxy variable, like volume, that could be used to estimate the relative numbers of the objects to be counted. Every other section is removed from the rank ordered series and added to the end of the series in reverse rank order. This will ideally increase the smoothness of the distribution for sampling, providing a relatively symmetric distribution around the midpoint. Especially when considering irregular objects, sampling from a series of sections arranged in this manner, rather than by their biological order, makes it less likely that final counts will be biased by selection of sections with unusually high or low particle densities.

## Anticipated Results

### Immunolabeling

**FIGURE 7:**
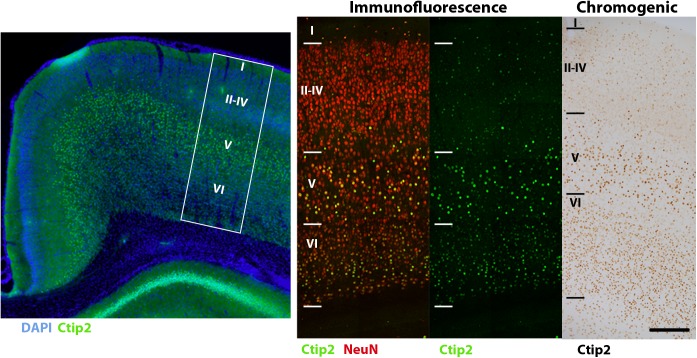
Comparison of Ctip2 labeling with immunofluorescence and chromogenic immunohistochemistry. Subcortical projection neurons of the neocortex (left) can be identified by labeling Ctip2 with AF-488 (green), NeuN with AF-647 (red), and nuclei with DAPI (blue) using immunofluorescence. White box demarcates representative location of Ctip2 and NeuN labeled panels to the right. Immunofluorescence produces clearer and more precise labeling than using the brown chromogen DAB (DAB Peroxidase Substrate Kit, Vector, SK-4100) to label the same rabbit anti-Ctip2 antibody for brightfield microscopy. Fluorophore signal was clearer and easier to discriminate from background labeling and co-labeling with NeuN helped to more clearly identify this neuronal subclass. Scale bar, 100 μm.

Our immunofluorescence protocol produced robust labeling of all our antigens of interest in the appropriate fluorescence channels. As most fixed tissue requires some degree of antigen retrieval, steamer-mediated antigen retrieval is arguably the most reliable, accessible technique (Ramos-Vara and Miller, 2014). All six primary antibodies tested followed their well-characterized labeling patterns. The markers used in our stereological analysis, Ctip2 and NeuN (Figure 2), were visible in the green and far red channels, respectively. Ctip2 labeling was predominant in neuronal nuclei in layer V but also evident in other layers, as might be anticipated particularly at this early developmental timepoint (Arlotta et al., 2005). We compared our fluorescence labeling of Ctip2 to enzymatic labeling with DAB using the same Ctip2 primary antibody. Fluorescence labeling resulted in reduced background relative to enzymatic techniques, making it easier to discriminate (Figure 7). NeuN expression followed its standard labeling patterning, localized to neuronal nuclei and immediately surrounding neuronal cytoplasm (Mullen et al., 1992). The majority of Ctip2^+^ cells were co-labeled with NeuN. In a different series, we were able to use all four channels with antibodies to S100β, Iba1, and Olig2, with DAPI in the blue channel, without worrying about bleed-through affecting cell identification due to the distinct labeling patterns of these markers (Figures 3A,C). In the far red channel, Olig2 expression in oligodendrocytes was nuclear and seen predominantly in the white matter, where the majority of oligodendrocytes reside (Zhou and Anderson, 2002). Antibodies to Iba1, in the green channel, and S100β, in the red channel, revealed cell bodies and processes of microglia and astrocytes, respectively—labeling cells that were tiled across the cortex and showed distinct cellular morphologies between gray and white matter (Dyck et al., 1993; Ito et al., 1998). In a separate series, tissue labeled with Iba1 in the green channel and CD68 in the red (Figure 3B) showed only a subset of microglia were CD68^+^; this was primarily along white matter tracts, where microglia are consistently reported to show higher expression of markers of activation (Harry and Kraft, 2012; Ling and Tan, 1974). CD68 expression was punctate, characteristic of its standard endosomal and lysosomal localization pattern. In all cases, background was low and cells were easily distinguishable.

**FIGURE 8:**
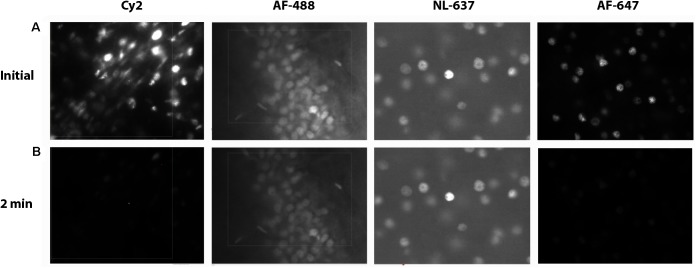
Fluorophore bleaching. Epifluorescent images of Ctip2^+^ cells at 60× at an initial exposure after ∼0.1 min **(A)** and after 2 min of exposure **(B)** with Cy2, AF-488, NL-637, and AF-647 secondary antibodies.

**FIGURE 9:**
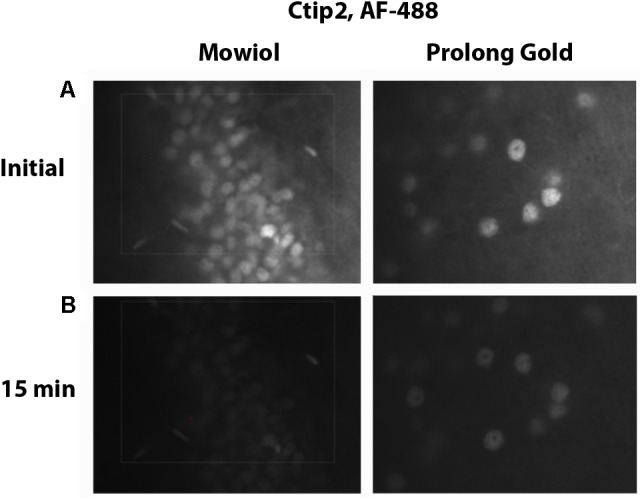
Mounting media bleaching. Tissue immunostained for Ctip2 with an AF-488 secondary antibody was mounted with Mowiol or Prolong Gold. Images show initial fluorescence **(A)**, and fluorescence after 15 min of exposure **(B)**.

Choosing the right fluorescence secondary antibody label is critical for stereological analysis. In our pilot study, we placed our two nuclear labels in the green, FITC, and far red, Cy5, channels which minimized bleed-through. However, the process of sampling cells induces photobleaching which could, additionally, bias final counts. Before starting the optical fractionator, we tested the resistance of several fluorophores to photobleaching using donkey anti-rabbit secondary antibodies conjugated to fluorophores expressing in green, Cy2 (Jackson) and AF-488 (Life Technologies), or far red, AF-647 (Jackson) and NL-637 (R&D Systems), channels (Figure 8). We found significant bleaching of Cy2 even within 30 s. AF-647 was also unstable, bleaching 72% after 2 min of exposure. AF-488 was much more stable, bleaching 36% over this same interval, and NL-637 was the most stable, bleaching only 2.9% (Table 2). In addition to fluorophore stability, the choice of mounting medium can help to minimize photobleaching. We compared bleaching of the less stable AF-488 fluorophore when coverslipping with Mowiol, a commonly used medium, and Prolong Gold (Thermo Fisher, P10144), developed to reduce bleaching (Figure 9). After 2 min of exposure, there was a 36% reduction in signal with Mowiol and 21% reduction with Prolong Gold (Table 2). Additionally, we find our fluorescent labeling to last for more than a year when stored in the dark at 4°C, allowing sufficient time for subsequent data collection or reliability testing.

**Table 2:** Photobleaching experiments.

	Mountant	Exposure duration (min)	Mean gray value	Signal reduction (%)
**AF-647**	*Mowiol*	0.1	6.1	–
		2	1.7	72.1
		15	0.0	100.0
**NL-637**	*Mowiol*	0.1	78.3	–
		2	76.0	2.9
		15	58.5	25.3
**Cy2**	*Mowiol*	0.1	33.8	–
		2	2.8	91.8
		15	0.0	100.0
**AF-488**	*Mowiol*	0.1	61.8	–
		2	39.5	36.1
		15	22.0	64.3
**AF-488**	*Prolong Gold*	0.1	56.7	–
		2	44.8	20.9
		15	31.8	43.9


*Various fluorophores and mounting media were tested for rates of photobleaching by measuring pixel intensity initially, at 2 min after exposure to replicate a sampling run, and at 15 min to attempt to measure total signal quenching.*

Balancing cost, availability, and efficacy, we found the best strategy was to use the common AlexaFlour (AF) line of fluorophores and the mountant Mowiol. This combination produced a green signal strong enough to reliably quantify our most difficult label, Ctip2. Because NeuN is robustly expressed and can be rapidly quantified, AF-647 was sufficient, despite its more rapid photobleaching. Of course, we did confirm that anti-fade mounting media and extremely stable fluorophores, such as the Northern Lights (NL) antibodies, exhibit decreased photobleaching rates. For some epitopes, it may be advisable to use the newer fluorophores which are marketed as being particularly photostable like the NL line. Prior to data acquisition, investigators should always perform pilot studies to ensure they are adequately familiar with the labeling patterns of the antigens of interest and the optimal fluorophores are chosen for their sampling scheme to minimize bias and time spent at each counting site. While our protocol was tested on perfusion-fixed tissue, we have found it to be effective using immersion-fixed tissue as well. Although immunolabeling may have to be optimized for variation in fixation and individual antibodies, we were able to identify diverse cell types with our protocol and anticipate it would reveal most epitopes.

### Stereology

**FIGURE 10:**
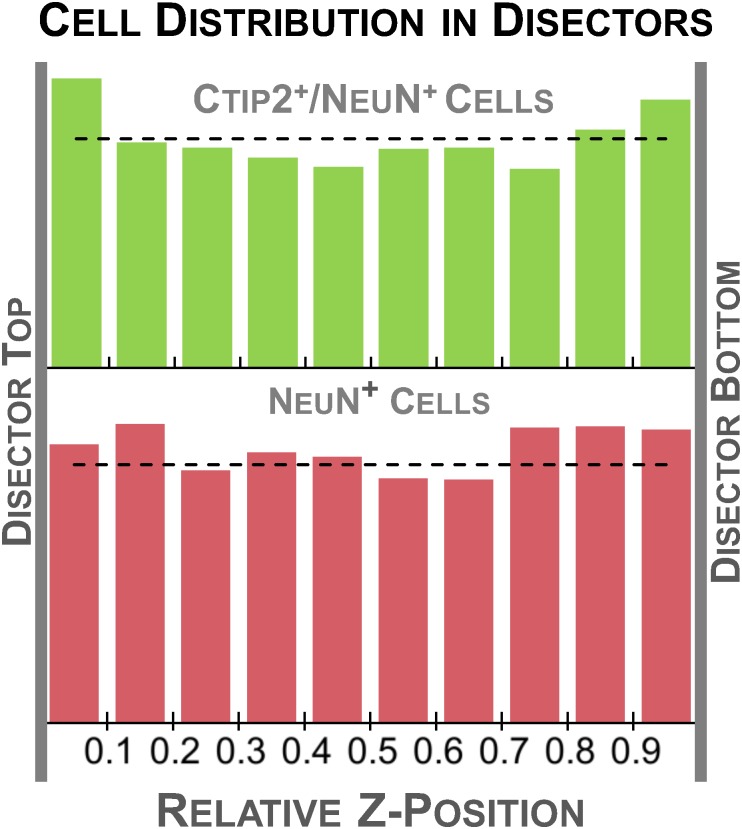
Histogram of relative z-depth in the disector for each cell type sampled. The dashed line indicates the average number of cells expected for each bin (total cells sampled/10 bins), representing a perfectly even distribution. Cells were relatively evenly distributed through the thickness of the tissue, suggesting good penetration of both our Ctip2 and NeuN antibodies. A slight U-shaped distribution can be observed in the Ctip2 graph. The higher density regions at the tissue margins could be avoided by decreasing the height of the disector and increasing the height of the guard zone.

To validate our protocol, we performed a stereological assessment of the numbers of neurons, defined by NeuN expression, and subcerebral projection neurons, defined by the additional expression of Ctip2, in rat cerebral cortex at postnatal day 10. The stereological parameters from our pilot study are summarized in Table 3, a representative table illustrating the minimum criteria necessary to report for optical fractionator analysis per Schmitz and Hof (2005). Tissue series produced with our immunolabeling protocol met the expectations of stereological analysis. Labeling was sufficiently bright and distinct from background to facilitate reliable identification of our criterion for inclusion, the nucleus at its widest point, in all sampled cells (Figure 2). The final average section thickness was ∼20 μm, preserving 40% of tissue height. This increased post-processing tissue thickness provides an advantage over enzymatic immunohistochemical techniques which produce more extensive shrinkage due to multiple dehydration steps. It allowed us to add generous 4.5 μm guard zones to either end of our disectors. Disectors were well within the standard range, 9 μm for neurons and 11 μm for subcerebral projection neurons. Analysis of the *z*-distribution within our disectors indicates even antibody penetration throughout its height (Figure 10). However, our pilot data suggest cells may be clustered more at the top and bottom of our larger 11 μm disector. This could reflect compression artifacts and might warrant decreasing the disector height and increasing guard zones for subsequent analyses to ensure a more even distribution.

**Table 3:** Stereological analysis parameters.

Variable	Ctip2	NeuN
Mean number of investigated sections	16	16
Mean number of investigated microscopic fields	328	159
Mean actual section thickness after histologic processing (μm)	21.3	18.3
Grid size (μm)	690 × 665	1005 × 925
Disector area (μm^2^)	1600	900
Disector height (μm)	11	9
Guard zone height (μm)	4.5	4.5
Mean number of counted cells	447	525
CE	0.05	0.04


*Summarized are the parameters that should be reported for all stereological studies. We have listed our final parameters for Ctip2 and NeuN from our pilot study (N = 5).*

**FIGURE 11:**
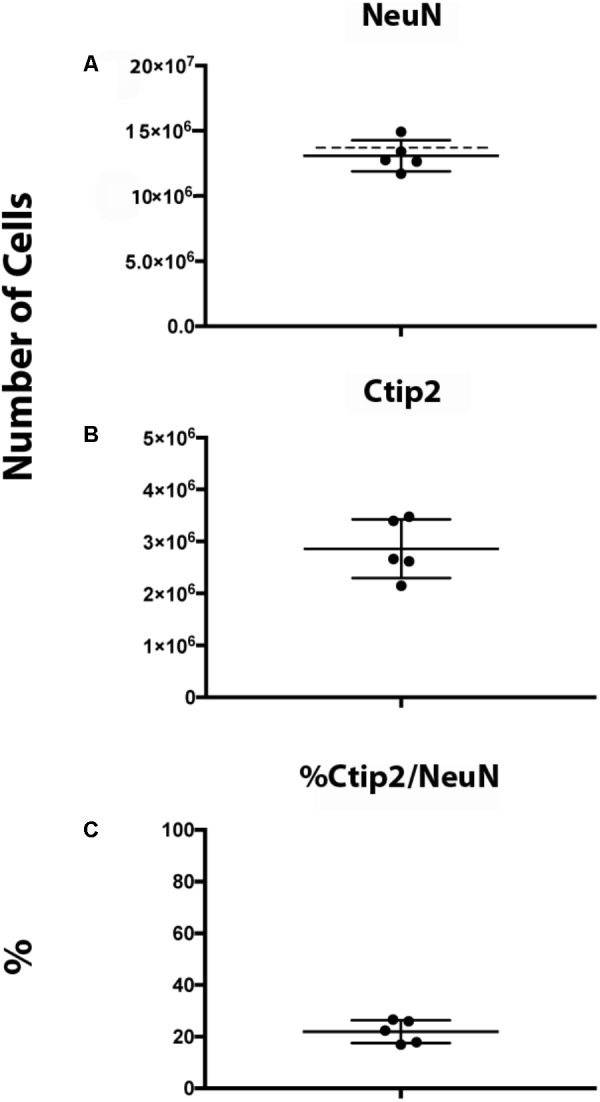
Ctip2 and NeuN quantification in postnatal day 10 rat neocortex. Dot plots show total numbers of NeuN^+^ **(A)** and Ctip2^+^ **(B)** neurons based on number-weighted section thickness. Dashed line shows estimate of NeuN^+^ neurons in postnatal day 11 rat cortex obtained by Bandeira et al. (2009). Percent of NeuN^+^ cells that are Ctip2^+^ is shown in **(C)**. Data represent mean and standard deviation (*N* = 5).

We obtained an estimate of 13.1 ± 1.2 million NeuN^+^ neurons and 2.8 ± 0.6 million Ctip2^+^/NeuN^+^ subcerebral projection neurons using our protocol (Figure 11). An average of 447 (325–553) neurons and 525 (413–590) subcerebral projection neurons were sampled. Our neuronal estimate correlates well with prior estimates obtained in rat of 14.4 ± 0.6 million neurons at P11 (Bandeira et al., 2009). The similarity in estimates is particularly notable considering the dynamic nature of this developmental period, when neuron production, maturation, and elimination cause rapid fluctuation in numbers (Lyck et al., 2007; Bandeira et al., 2009), and that Bandeira and colleagues employed a different method, the isotropic fractionator. Neuron numbers in mice at a similar developmental stage are lower, ∼6 million, as can be expected based on evolutionary scaling in brain size and neuron proportions (Lyck et al., 2007; Herculano-Houzel et al., 2010; Karacay et al., 2015). To our knowledge, this is the first quantification of Ctip2^+^ neurons that has been performed in the postnatal rat brain. Subcerebral projection neurons comprised 22% of total neurons. Across the sample, we found that the variance in this percentage was low, indicating strong internal consistency. For both markers, our estimates yielded low Gundersen CEs (*m* = 1), 0.042 for NeuN and 0.050 for Ctip2, indicating low sampling error. In comparison, the CV was higher, 0.091 for NeuN and 0.20 for Ctip2, making the error introduced by stereological design (CE) less than 50% of the total variance, as recommended (Gundersen et al., 1999). In addition, our intrarater reliability was high, 0.997 for NeuN and for 0.977 Ctip2, attesting to the ease of identifying cellular subtypes with this protocol.

## Conclusion

We hope that the high reliability estimates we have obtained through application of a relatively simple immunostaining protocol illustrate the accessibility of this gold standard cell quantification technique to researchers in neuroscience. It is increasingly easy to obtain high quality, well vetted antibodies, and an array of fluorophores have been and continue to be designed for enhanced stability. Modification of existing stereology systems for immunofluorescence can be accomplished on relatively modest budgets, especially given the advent of newer systems using inexpensive and long-lasting LED light sources. These factors make it increasingly feasible to incorporate multiple immunofluorescence into stereological design to answer more complex questions more precisely, substantially increasing the explanatory power of individual experiments. Combining the statistical rigor of stereological sampling with the increased precision of immunofluorescence can provide the technical improvements needed to explore novel questions and refine existing ones.

## Author Contributions

NB and AK designed the experiments, wrote the manuscript, collected the images, and revised the manuscript. AK executed experiments.

## Conflict of Interest Statement

The authors declare that the research was conducted in the absence of any commercial or financial relationships that could be construed as a potential conflict of interest.
